# Major breakthroughs in hematopoietic stem cell transplantation and future challenges in clinical implementation

**DOI:** 10.1172/JCI179944

**Published:** 2024-04-15

**Authors:** Leslie S. Kean, Bruce R. Blazar

**Affiliations:** 1 Division of Pediatric Hematology and Oncology, Boston Children’s Hospital and the Dana-Farber Cancer Institute; Department of Pediatrics, Harvard Medical School, Boston, Massachusetts, USA.; 2 Department of Pediatrics, Division of Blood & Marrow Transplant & Cellular Therapy, and the Masonic Cancer Center, University of Minnesota, Minneapolis, Minnesota, USA.

The Haitian proverb “Beyond mountains, there are mountains” has many meanings. One that resonates for hematopoietic stem cell transplantation (HCT) over the last 50-plus years is the sense that as you solve one problem, another presents itself. To mark the 100th anniversary of the *JCI*, we focus on the evolving understanding of major HCT hurdles, those surmounted, challenges that lie ahead (Figure 1), and how these have been chronicled through key *JCI* papers.

## Hematopoietic stem cell transplantation

Hematopoietic stem cell transplantation (HCT) is a curative therapy for many malignant and nonmalignant hematologic diseases, including treatment-refractory leukemia, classical hematologic diseases (e.g., sickle cell disease), congenital and acquired immune system disorders (e.g., severe combined immune deficiency), and inborn errors of metabolism (e.g., Hurler syndrome). Allogeneic HCT refers to a transplant where donor BM, peripheral blood–based stem cell, or umbilical cord blood products are infused into a patient after myeloablative or nonmyeloablative conditioning regimens involving irradiation and/or chemotherapy; these are designed to make room for donor stem cells, immune suppress the host to permit donor graft acceptance, and eliminate residual malignant cells. Autologous HCT refers to infusion of the patient’s own stem cells to restore hematopoiesis. Despite considerable successes, multiple challenges still loom. For allogeneic HCT, the focus of this Viewpoint, the greatest challenges are relapse of the primary disease and graft-versus-host disease (GVHD). Each deserves special discussion given their strong connection to the immunology of HCT, as well as to other HCT complications, including infection, graft failure, and organ failure.

For patients with malignant hematologic diseases, the transplanted donor hematopoietic stem and progenitor cells serve two purposes: (i) they replace the diseased BM; and (ii) they protect against relapse by what is known as the graft-versus-leukemia (GVL) effect, whereby transplanted immune cells, including both T cells and NK cells, provide immunologic surveillance and clearance of malignant cells. One of the greatest challenges of HCT is that the T cells that are so important for immune reconstitution and for GVL are also the major mediators of GVHD. Acute and chronic GVHD occur when T cells (along with other immune cells) fail to achieve immune tolerance to the tissues and organs of the recipient, become activated and pathogenic, and cause immune-mediated tissue destruction. During acute GVHD (aGVHD), immune cells cause inflammatory tissue damage to canonical organs (including the gastrointestinal [GI] tract, liver, and skin) along with other tissues (e.g., lung, CNS) (1). During chronic GVHD (cGVHD), T cells orchestrate a complex pathogenic program that also involves B cells and other immune mediators to cause sclerotic, fibrotic, and/or inflammatory damage, which can target any tissue in the patient and is the major cause of morbidity and mortality in patients surviving beyond 100 days after HCT (2).

## Progress and key hurdles in allogeneic HCT

A landmark 1959 article by Thomas et al. reported the first patients with leukemia given lethal doses of irradiation followed by HCT with bone marrow donated by the patients’ identical twins; this treatment overcame the alloengraftment barrier, circumvented GVHD barriers, and presciently highlighted critical antileukemia therapeutic needs (e.g., GVL effects) (3). Not until 1971 would serotyping and mixed lymphocyte culture testing of canine siblings confirm HLA matching and definitively show that without immune suppression, minor histocompatibility (miH) antigen mismatches pose a substantial risk of GVHD lethality (4), setting the stage for pursuing GVHD antigen discovery and predictive, diagnostic, and prognostic biomarkers (see below). Rapid, long-term hematopoietic recovery provided indirect evidence for chimerism and implied that bona fide donor hematopoietic stem cells (HSCs) repopulated the BM niche. Decades later, Ginsburg et al. deployed a sensitive, multilineage assay using DNA polymorphisms to quantify donor and host chimerism levels (5).

## Identifying CD34^+^ HSCs ushered in graft engineering

Many studies provided circumstantial evidence that the elusive HSC resided in a BM CD34^+^ subset. In a tour-de-force study using a cross-reactive nonhuman primate and human anti-CD34 mAb, Berenson et al. showed that CD34-enriched but not CD34-depleted cells are engraftable (6). The ability to manipulate CD34^+^ cells, a definition that became synonymous with HSCs, led to efforts to increase CD34^+^ cell numbers to achieve rapid hematopoietic recovery and to utilize this population for stem cell gene therapy. Cord blood CD34^+^ cells incubated with small-molecule nicotinamide, a primary precursor of nicotinamide adenine dinucleotide (NAD^+^) and cofactor in multiple redox reactions, showed promise for ex vivo HSC expansion, as reported in 2014 (7). With continued development, nicotinamide treatment secured FDA approval for stem cell therapy in 2023.

In a clinical trial, Bleakley, Shlomchik, and colleagues employed donor graft CD34^+^ selection supplemented with T cells depleted of GVHD-causing naive cells and enriched in memory cells that have the capacity to mediate antitumor and antimicrobial immunity (8). This strategy was hypothesized to decrease both aGVHD and cGVHD; cGVHD was virtually eliminated, but surprisingly, aGVHD frequency was unchanged. These data, indicative of the distinct biology of the two GVHD counterparts, offered a platform for cellular therapies under conditions wherein there is a prerequisite for enrolling cGVHD-free patients for safety and efficacy evaluation.

## Transformation of GVHD prophylaxis by posttransplant cyclophosphamide

Despite improved HCT outcomes, GVHD remains a difficult mountain to climb. Notable successes and new hurdles have arisen with each advance. We cannot discuss GVHD prevention without first highlighting the seismic change in the field, based upon decades of pioneering studies by Johns Hopkins investigators, that incorporated posttransplant cyclophosphamide (PT-Cy) into a commonly used GVHD prophylaxis regimen. Adding PT-Cy to tacrolimus and mycophenolate mofetil (MMF) resulted in a striking decrease of cGVHD (9), similar to naive T cell depletion. Compared with the historic standard of care of a calcineurin inhibitor plus methotrexate for GVHD prophylaxis, PT-Cy/tacrolimus/MMF led to a significantly improved composite cGVHD survival endpoint (Bone Marrow Transplant Clinical Trials Network trial [BMT CTN 1703]).

It has been challenging to rigorously pin down the mechanism(s) by which PT-Cy prevents cGVHD in patients. Furthermore, there are substantial trade-offs with this therapy, including the cardiac toxicity inherent to cyclophosphamide, as well as adverse effects on T cell reconstitution. Toward understanding both the benefits and risks of PT-Cy, Kanakry’s group has pursued elucidation of the mechanisms by which PT-Cy prevents GVHD in mice (10). They found that GVHD prevention by PT-Cy involved complex immunologic mechanisms including T cell dysfunction and increased suppression associated with rapid reconstitution of Tregs. Whether these mechanisms can be observed in humans is an open question and one that is currently being answered through the BMT CTN 1801 biology study (ClinicalTrials.gov NCT03959241).

## Inactivation of GVHD-causing T cells by costimulatory blockade

Rather than nonspecific removal or incapacitation of donor T cells, a long-standing GVHD prophylaxis goal has been the targeted inactivation of GVHD-causing T cells. Blockade of T cell costimulatory signals, designed to selectively prevent alloreactivity while retaining protective immunity, inspired our group to investigate multiple costimulatory pathways, with a special focus on CTLA-4–Ig (abatacept) and anti-CD28 mAb blockade of CD28-CD80/86 costimulation (11). These studies provided an important counterpart to the PT-Cy story, wherein abatacept has proven highly effective in preventing aGVHD, but not cGVHD (at least with a 4-dose regimen). Abatacept’s substantial effects of decreasing aGVHD and improving survival after unrelated-donor HCT led to its being the first FDA-approved GVHD prevention strategy. A 4- versus 8-dose randomized trial geared toward preventing both aGVHD and cGVHD (ClinicalTrials.gov NCT04380740) underscores the critical need for clinical trials to investigate tradeoffs we now face to identify regimen(s) that functionally controls aGVHD and cGVHD without physical T cell depletion.

A promising partner to CD28-80/86 blockade is the CD40:CD154 pathway. A classic paper by Blazar et. al first documented anti-CD154 mAb–induced pre-HCT donor CD4^+^ T cell alloantigen-specific tolerization and protective immunity retention (12). Long-lived, effective anti-CD154 mAbs that do not cause unwanted thrombotic complications now exist and have advanced to clinical trials for ameliorating autoimmune disease and preventing solid organ graft rejection.

## Allorecognition of host antigens in GVHD and autoimmunity

As the field advances with increasingly efficacious therapeutics, the quest to understand mechanisms causing GVHD and GVHD-inciting host antigens continues. Korngold found that CD4^+^ T cell allorecognition of multiple miH antigens exclusively presented on hematopoietic cells resulted in self-limited aGVHD, whereas lethal aGVHD was induced by nonlymphoid miH antigenic disparities (13). Maillard’s lab added to this puzzle by proving that GVHD-amplifying signals can be delivered by host fibroblastic stromal cells including fibroblastic reticular cells (FRCs) that express Notch ligands and are juxtaposed to alloreactive T cells (14). As described by Chakraverty et al., GVHD downregulated expression of the autoimmune regulator–like transcription factor DEAF1 — necessary for intranodal peripheral tissue–restricted self-antigen display and prevention of autoimmunity — in FRCs. Ultimately, FRC depletion by GVHD may account for the acute-to-chronic GVHD transition (15).

## Restoring immune homeostasis with Treg infusion

A major step forward in our understanding of cGVHD mechanisms was provided by Ritz and colleagues, who observed inadequate peripheral reconstitution of naive Tregs caused by GVHD-induced thymus injury, resulting in a predominance of proliferating activated/memory Tregs with high susceptibility to Fas-mediated apoptosis and failed cGVHD control of autoimmune-like manifestations (16). Simultaneous with studies by Edinger et al. (17), Cohen’s lab showed that recipient-specific Tregs not only controlled GVHD and GVL effects but facilitated immune reconstitution (18).

## Strategies to overcome tissue injury in HCT

As the field of GVHD mechanistic studies has matured and new strategies are sought to continue to perfect GVHD prophylactic regimens, the focus of the field has turned increasingly to understanding drivers of tissue injury. Contrary to the long-accepted paradigm that donor T cells alone cause aGVHD tissue destruction, Kupper and colleagues published their finding that proliferating, proinflammatory T cells that were 100% of host origin could be found adjacent to donor antigen-presenting cells (APCs) in patient with skin aGVHD, consistent with host skin-resident T cell activation by donor APCs and subsequent tissue injury (19). Key data pertinent to GI tract injury are findings of increased levels of circulating regenerating islet-derived protein III-gamma (Reg3γ), an antimicrobial peptide released by damaged intestinal Paneth cells, coincident with this deadly GVHD manifestation (20). Such observations led to the administration of IL-22, produced by gut innate lymphoid cells type 3 (ILC3s) to stimulate nonhematopoietic epithelial and stromal cell proliferation, host defense at barrier surfaces, and intestinal stem cell proliferation, resulting in increased Paneth cell and decreased Reg3γ levels.

Antiinflammatory gut ILC2s can ameliorate GI tract damage, as shown by Bruce, Serody, and colleagues, who demonstrated marked intestinal ILC2 depletion following a pre-HCT conditioning regimen that required at least 3 months for repopulation (21). Conversely, the infusion of donor ILC2s, isolated from the peritoneum, mitigated aGVHD lethality due at least in part to IL-13–stimulated myeloid-derived suppressor cells.

## Chronic GVHD therapies achieve FDA approval at last

As allo-HCT has become safer overall, clinical trials have increasingly included older patients who receive peripheral blood stem cell grafts. However, these transplant paradigms can increase cGVHD risk, which emphasizes the need for continued preclinical investigations into the mechanisms of cGVHD prevention and treatment as a prelude to clinical translation. Collaborative studies between the Blazar and Byrd laboratories showed that the dual Bruton’s tyrosine kinase (BTK)/IL-2–induced T cell kinase (ITK) inhibitor ibrutinib could reduce murine cGVHD, including in preclinical models of often treatment-refractory lung disease (bronchiolitis obliterans) and sclerotic skin manifestations (22). A phase I ibrutinib trial in patients with steroid-refractory cGVHD designed to preclude pathogenic B cell–T cell cooperativity led to rapid FDA approval for this debilitating disease. Other FDA-approved immunomodulatory drugs for steroid-refractory or -resistant cGVHD include inhibitors of the Rho-associated coiled-coil containing protein kinase 2 (Rock-2; belumosudil) (23) and JAK1/2 (ruxolitinib) (24).

Groundbreaking studies by MacDonald, Blazar, Hill, and colleagues identified another major cellular contributor to cGVHD: profibrotic macrophages recruited to the skin and the lung, regions with antibody deposition and tissue injury, and implicated in scleroderma-like skin and lung bronchiolitis obliterans (25). Neutralizing anti-CSF1R mAb efficacy in cGVHD mice has now been successfully translated into the clinic to treat refractory cGVHD (ClinicalTrials.gov NCT04710576).

## Toward a nontoxic pre-HCT conditioning regimen

One of the major hurdles that remains in our field is the development of less-toxic but highly effective targeted approaches to engraftment during HCT. Toward this goal, the DiPersio lab has described antibody-drug conjugates (ADCs) against CD45 and CD117 that are able to effectively clear the BM and enable alloengraftment across MHC barriers, a major advance (26). When ADCs were combined with JAK1/2 inhibitors and BM ablation, alloengraftment across MHC barriers was achieved, GVHD was ameliorated, GVL was maintained, and thymic function was preserved, highlighting the next horizon of combined targeted conditioning and immune modulation for optimal patient safety and efficacy.

## The uphill challenge of controlling GVHD and post-HCT relapse

Strategies to control aGVHD and cGVHD, restore immune homeostasis, and minimize myeloablative regimen toxicity, each can contribute to an increased risk of relapse after HCT. On the other hand, proactive approaches to remove GVHD-causing donor graft T cells while sparing donor cells that possess GVL properties (8) or administering GVHD prophylaxis or treatment agents that spare steroids can inhibit aGVHD (26) and cGVHD (22) and even eradicate leukemia cells, thereby actually augmenting GVL. Toward these goals, seminal studies by the Murphy laboratory demonstrated that after infusion, activated NK cells had the anti-GVHD and pro-GVL effects of killing tumor cells and, via TGF-β release, mitigating aGVHD (27). Subsequently, Romee and colleagues infused in vitro generated cytokine-induced memory-like NK cells and reported a rapid, 10- to 50-fold expansion and increased in vivo cytolytic potency, along with a reduction in the level of malignant myeloid-lineage cells in 4 of 6 patients (28). Betts and Davila eradicated human CD83^+^ pathogenic CD4^+^ T cells, proinflammatory DCs, and AML cells by redirecting donor T cells to express an anti-CD83–expressing chimeric antigen receptor (29), adding another dual-purpose immune-based GVHD and GVL strategy. These examples highlight the risks, and new potential solutions, to the major problem of malignant disease relapse that continues to loom after transplant.

## Conclusion

Though we have yet mountains to climb, this 50-year journey, viewed through the lens of *JCI* publications, illustrates remarkable progress and serves as a guidepost and North Star for future accomplishments.

## Figures and Tables

**Figure 1:**
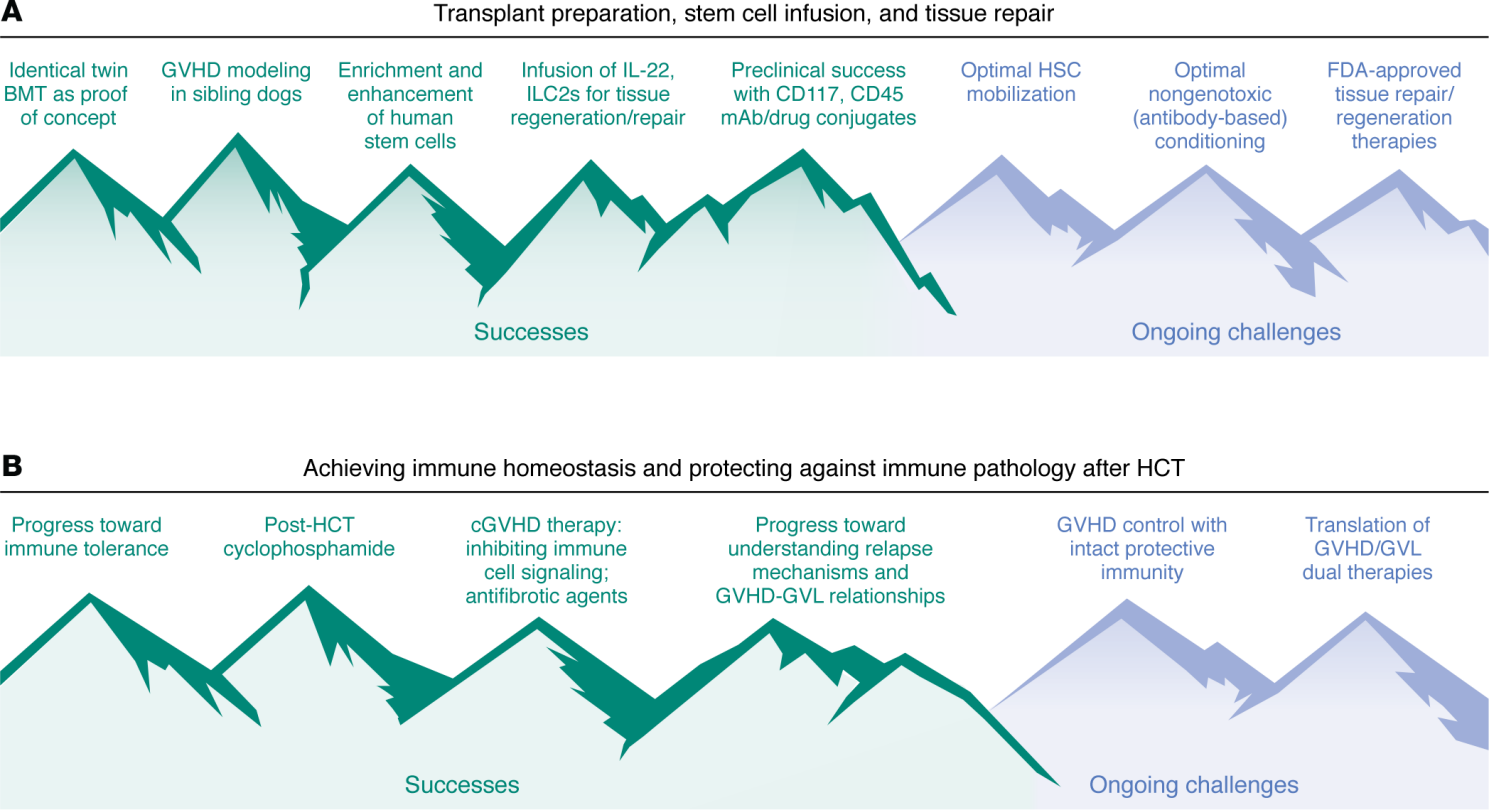
Mountain climbing: key advances in HCT and the unsolved challenges remaining. (**A**) Key accomplishments (left) and challenges remaining (right) for transplant preparation (3, 4), stem cell infusion (6, 7), and tissue repair after HCT (20, 21, 26). (**B**) Key accomplishments and challenges remaining for achieving immune homeostasis (11–18) and protecting against immune pathology after HCT (8–10, 22–29). References highlight major *JCI* articles contributing to the described discoveries.

## Version 1 04/15/2024

Electronic publication

## References

[1] Zeiser R, Blazar BR (2018). Acute graft-versus-host disease. N Engl J Med.

[2] Zeiser R, Blazar BR (2017). Pathophysiology of chronic graft-versus-host disease and therapeutic targets. N Engl J Med.

[3] Thomas ED (1959). Supralethal whole body irradiation and isologous marrow transplantation in man. J Clin Invest.

[4] Storb R (1971). Marrow grafts between canine siblings matched by serotyping and mixed leukocyte culture. J Clin Invest.

[5] Ginsburg D (1985). Origin of cell populations after bone marrow transplantation. Analysis using DNA sequence polymorphisms. J Clin Invest.

[6] Berenson RJ (1988). Antigen CD34+ marrow cells engraft lethally irradiated baboons. J Clin Invest.

[7] Horwitz ME (2014). Umbilical cord blood expansion with nicotinamide provides long-term multilineage engraftment. J Clin Invest.

[8] Bleakley M (2015). Outcomes of acute leukemia patients transplanted with naive T cell-depleted stem cell grafts. J Clin Invest.

[9] Bolanos-Meade J (2023). Post-transplantation cyclophosphamide-based graft-versus-host disease prophylaxis. N Engl J Med.

[10] Wachsmuth LP (2019). Post-transplantation cyclophosphamide prevents graft-versus-host disease by inducing alloreactive T cell dysfunction and suppression. J Clin Invest.

[11] Watkins BK (2018). CD28 blockade controls T cell activation to prevent graft-versus-host disease in primates. J Clin Invest.

[12] Blazar BR (1998). CD4(+) T cells tolerized ex vivo to host alloantigen by anti-CD40 ligand (CD40L:CD154) antibody lose their graft-versus-host disease lethality capacity but retain nominal antigen responses. J Clin Invest.

[13] Jones SC (2003). Importance of minor histocompatibility antigen expression by nonhematopoietic tissues in a CD4+ T cell-mediated graft-versus-host disease model. J Clin Invest.

[14] Chung J (2017). Fibroblastic niches prime T cell alloimmunity through Delta-like Notch ligands. J Clin Invest.

[15] Dertschnig S (2020). Graft-versus-host disease reduces lymph node display of tissue-restricted self-antigens and promotes autoimmunity. J Clin Invest.

[16] Matsuoka K (2010). Altered regulatory T cell homeostasis in patients with CD4+ lymphopenia following allogeneic hematopoietic stem cell transplantation. J Clin Invest.

[17] Edinger M (2003). CD4+CD25+ regulatory T cells preserve graft-versus-tumor activity while inhibiting graft-versus-host disease after bone marrow transplantation. Nat Med.

[18] Trenado A (2003). Recipient-type specific CD4+CD25+ regulatory T cells favor immune reconstitution and control graft-versus-host disease while maintaining graft-versus-leukemia. J Clin Invest.

[19] Divito SJ (2020). Peripheral host T cells survive hematopoietic stem cell transplantation and promote graft-versus-host disease. J Clin Invest.

[20] Zhao D (2018). Survival signal REG3α prevents crypt apoptosis to control acute gastrointestinal graft-versus-host disease. J Clin Invest.

[21] Bruce DW (2017). Type 2 innate lymphoid cells treat and prevent acute gastrointestinal graft-versus-host disease. J Clin Invest.

[22] Dubovsky JA (2014). Ibrutinib treatment ameliorates murine chronic graft-versus-host disease. J Clin Invest.

[23] Cutler C (2021). Belumosudil for chronic graft-versus-host disease after 2 or more prior lines of therapy: the ROCKstar Study. Blood.

[24] Zeiser R (2021). Ruxolitinib for glucocorticoid-refractory chronic graft-versus-host disease. N Engl J Med.

[25] Alexander KA (2014). CSF-1-dependant donor-derived macrophages mediate chronic graft-versus-host disease. J Clin Invest.

[26] Persaud SP (2021). Antibody-drug conjugates plus Janus kinase inhibitors enable MHC-mismatched allogeneic hematopoietic stem cell transplantation. J Clin Invest.

[27] Asai O (1998). Suppression of graft-versus-host disease and amplification of graft-versus-tumor effects by activated natural killer cells after allogeneic bone marrow transplantation. J Clin Invest.

[28] Shapiro RM (2022). Expansion, persistence, and efficacy of donor memory-like NK cells infused for posttransplant relapse. J Clin Invest.

[29] Shrestha B (2020). Human CD83-targeted chimeric antigen receptor T cells prevent and treat graft-versus-host disease. J Clin Invest.

